# Prognostic effect of sidedness in early stage versus advanced colon cancer

**DOI:** 10.1002/hsr2.54

**Published:** 2018-06-27

**Authors:** H.F. Kennecke, Y. Yin, J.M. Davies, C.H. Speers, W.Y. Cheung, R. Lee‐Ying

**Affiliations:** ^1^ Medical Oncology Virginia Mason Cancer Institute Seattle WA USA; ^2^ Department of Medicine University of British Columbia Vancouver BC Canada; ^3^ Gastrointestinal Cancer Outcomes Unit British Columbia Cancer Agency Vancouver BC Canada; ^4^ Medical Oncology British Columbia Cancer Agency Vancouver BC Canada; ^5^ Department of Oncology University of Calgary Calgary Alberta Canada; ^6^ Tom Baker Cancer Centre Calgary Canada

**Keywords:** colon cancer, left‐sided, prognostic factors, right‐sided, stage

## Abstract

**Background and Aims:**

The prognostic effect of sidedness in colorectal cancer has been evaluated in numerous prospective and retrospective cohorts. Most of these have reported overall survival data; there is scant relapse‐free survival data in early stage disease. This study aimed to determine the effect of tumor sidedness in survival in early stage and relapsed colon cancer.

**Methods:**

Patients with stage I‐III colorectal cancer were identified from the BC Cancer Agency Gastrointestinal Cancer Outcomes Unit. Survival analysis by stage and sidedness was compared with the log‐rank test. Baseline characteristics were controlled by multivariate Cox‐proportional hazard models. In relapsed patients, bevacizumab and EGFR inhibitor (EGFRI) treatments were included and tested for interaction.

**Results:**

Among 5378 patients with stage I‐III colon cancer, patients with right‐sided stage II tumors experienced better relapse‐free survival compared with those with left‐sided tumors; right‐sidedness was not prognostic for RFS in stage III disease. When survival was considered in patients who relapsed, right‐sided tumors had inferior survival after relapse in both stage II and stage III tumors. At relapse, right‐sided outcomes were inferior regardless of biologic therapy. An interaction test revealed a significant association between sidedness and survival with EGFRIs.

**Conclusions:**

In this large, population‐based study, right‐sided presentation has a significant prognostic impact: in early stage, right‐sidedness is favorably prognostic among stage II tumors and not prognostic in stage III disease. After relapse, right‐ sidedness is associated with an inferior prognosis, regardless of initial stage of presentation. Colon tumor sidedness is independently prognostic and may be considered in treatment assignment for both early stage and advanced disease.

**Abbreviations**
AC: adjuvant chemotherapy
CCa: colon cancer
cf: cell free
CI: confidence interval
CIMP: Cpg Island Methylator Phenotype
CMS: consensus molecular subtypes
CT: Chemotherapy
DFS: Disease Free Survival
dMMR: defective mismatch repair
ECOG: Eastern Cooperative Oncology Group (ECOG)
EGFR: Epidermal Growth Factor Receptor
GICOU: Gastrointestinal Cancer Outcomes Unit (GICOU)
HR: hazard ratio
IQR: inter quartile range
L: Left‐Sided
LVI: lymphovascular invasion
MMR: mismatch repair
mOS: median overall survival
NR: Not reached
ORR: overall response rate
OS: overall survival
pMMR: preserved mismatch repair
PFS: progression‐free survival
PNI: perineural invasion
R: right‐sided
RFS: relapse‐free survival
SAR: survival after relapse
wt: wild‐type

## 1 BACKGROUND

Multiple studies have demonstrated differences between right and left‐sided colon cancer (CCa) in terms of clinical characteristics, pathology, and prognosis. Patients diagnosed with right‐sided CCa are more likely to be female, older, and have less acute presentation and more locally advanced tumors.^1^, ^2^ Right‐sided tumors are characterized by a higher rate of poor differentiation and genetic changes, including microsatellite instability (MSI), BRAF, and hypermethylation (CIMP).^3^, ^4^ While the prognostic effect of sidedness has been evaluated in numerous prospective and retrospective cohorts,^1^, ^5^, ^6^ most studies have reported overall survival (OS) data only,^1^, ^2^, ^5^, ^7^, ^8^ with very little data on Disease Free Survival (DFS) in early stage disease. If tumor sidedness is to be a useful as a treatment factor, then the effect must be well described in each of the different treatment settings of stage II, III, and IV/relapsed disease.

In the stage IV or relapsed setting, right‐sided colonic tumors have undeniably worse outcomes when compared with left‐sided ones. Subgroup analyses of randomized clinical trials consistently show inferior outcomes in right‐ versus left‐sided tumors.^9^, ^10^ Population‐based analyses have demonstrated similar results, with the hazard ratios (HR) for right‐sided disease ranging from 1.12 to 1.25 and showing statistical significance, when compared with left‐sided advanced disease.^2^, ^7^, ^11^

The prognostic value of sidedness is much less conclusive in the early stage I‐III setting. In a meta‐analysis of 1 437 846 patients, left CCa conferred a favorable pooled prognostic effect on OS [HR 0.82 (95% CI, 0.79‐0.84) *P* < 0.001], and this effect was independent of stage.^12^ By contrast, 3 studies of the population‐based SEER database showed that right‐sidedness confers a favorable prognostic effect in stage II CCa (HRs 0.91, 0.92, and 0.89), and only right‐sided stage III tumors had inferior OS (HRs 1.06, 1.12, 1.12).^5^, ^7^, ^8^ A limitation of these studies is that they did not include data on Relapse Free Survival (RFS) or more extensive prognostic factors.^2^, ^5^, ^8^, ^13^, ^14^ This is illustrated by a SEER propensity score‐matched analysis that found no difference in OS between right and left sided stage III tumors.^15^The data points to a dichotomous effect of sidedness, with right‐sided tumors faring slightly better in early, stage II presentation, but conferring a substantially inferior prognosis among patients with relapsed or stage IV presentation. Very recent studies have shed more light on this and failed to demonstrate a prognostic effect of sidedness in stage III for DFS.^16^, ^17^ The effect of sidedness in stage III disease is unclear and needs to be better described if sidedness is to be used as a prognostic factor.

In this study, our hypothesis was that the prognostic effect of tumor sidedness is different in early‐stage versus relapsed colorectal cancer, and the endpoints chosen were Relapse Free Survival (RFS) and Survival after Relapse (SAR). We reviewed demographic and prospectively collected outcome information from patients enrolled in the British Columbia Cancer Agency (BCCA) Gastrointestinal Cancer Outcomes Unit (GICOU) to establish the independent prognostic value of sidedness in CCa, determine the effect of sidedness according to stage of presentation of CCa, and explore the impact of bevacizumab and anti‐Epidermal Growth Factor Receptor inhibitor (EGFRi) therapy in right‐ versus left‐sided CCa in relapsed disease.

## 2 METHODS

### 2.1 Description of the study population

The BCCA is a province‐wide agency that provides comprehensive cancer care, including prevention, screening, diagnosis, and treatment to the residents of British Columbia, Canada. All systemic therapy is centrally reimbursed and documented in the Provincial Chemotherapy Database. The BCCA GICOU prospectively compiles demographic, diagnostic, treatment, and outcome data for all patients referred to the BCCA with gastrointestinal malignancies. Consent is obtained from all patients referred to BCCA for treatment delivery and to prospectively follow outcomes. An active follow‐up program including annual letters to primary care providers after discharge results in an estimated loss to follow‐up of <5%.

The GICOU database was used to identify all patients diagnosed with stage I, II, or III CCa in 1990, 1995, 1996, and 1999‐2009, and referred to any one of the BCCA centers for treatment of newly diagnosed CCa. The study time period was chosen to allow for sufficient follow‐up for reliable ascertainment of 5‐year survival. Specific cohorts were selected based on the availability of prospectively collected data.

Patients with a synchronous or prior CCa or a histological diagnosis other than adenocarcinoma were excluded, as were patients who relapsed within 4 months of diagnosis of early stage disease. Patients with tumors of unknown sidedness were excluded. Tumors were classified as right‐sided if they were of cecal, hepatic flexure, or transverse origin, while splenic flexure to sigmoid tumors were classified as left‐sided. Tumors within 16 cm of the anal verge were classified as rectal and were not included. Follow‐up data regarding survival and recurrence was gathered from visits to BCCA centers and through mail‐outs to the family physicians and surgeons of patients no longer regularly attending BCCA appointments. The BCCA‐UBC Research Ethics Board approved this study.

### 2.2 Definitions of dependent and independent variables

The functional status of patients was characterized with Eastern Cooperative Oncology Group (ECOG) performance status and was ascertained from the patient's records within 1 month of referral. Staging was based on the AJCC 6th Edition Cancer Staging Manual.^18^ Patients were deemed to have undergone a surgical resection if they had a surgery performed with curative intent and without gross (R2) residual disease. Patients were categorized as having received adjuvant chemotherapy if they completed more than 1 cycle of fluoropyrimidine‐based chemotherapy following curative resection. Information about whether patients with relapsed disease were treated with bevacizumab, cetuximab, and/or panitumumab was obtained from the BCCA Provincial Chemotherapy Database.

### 2.3 Statistical analysis

Baseline characteristics of the patients were summarized by sidedness. Categorical and continuous characteristics were compared, respectively, using Chi‐square test and Wilcoxon‐rank sum test. Kaplan Meier survival analysis was performed by stage, and the impact of sidedness compared with the log‐rank test. The patients are censored at 10‐year of follow‐up when RFS were calculated. RFS was measured from date of surgery until local, regional, or distant recurrence, and censored at death or last follow‐up or 10‐year if follow‐up was longer than 10 years. In the subset of patients who relapsed, SAR was measured from the documented date of local, regional or distant relapse, until death or last follow‐up. Cox‐proportional hazard models were used to control for baseline clinicopathologic characteristics, including age at diagnosis, sex, grade, lymph node sampling, lymphovascular invasion, perineural invasion, performance status, and receipt of adjuvant chemotherapy. When the proportional hazard assumption was violated by any baseline clinicopathologic characteristics, the Cox‐proportional hazard model was stratified on those that violated the assumption. For bevacizumab and cetuximab or panitumumab after relapse, their interaction with sidedness was built respectively in the Cox model, to test for the association of survival with treatment and sidedness. When the proportionality assumption of the Cox regression was violated for a chemotherapy treatment, an interaction of this treatment with time was included in the model. An alpha level of 0.05 (2‐sided) was used for all statistical tests. For the KM univariate survival analysis, SPSS v.14 was used. The remainder of analyses were done using SAS v.9.4.

## 3 RESULTS

### 3.1 Patient demographics.

A total of 5358 patients with stage I‐III resected CCa, diagnosed between 1990 and 2009, were identified, and baseline characteristics described (Table 1); 46% of patients had right‐ and 54% had left‐sided cancer.

**Table 1:** Baseline characteristics of 5378 patients with stage I‐III colon cancer according to side of presentation

Characteristics	Right *N* = 2465 (46%)	Left *N* = 2913 (54%)	Significance Chi‐Square
Age			
Median (IQR)	70 (61‐77)	66 (58‐79)	*P* < 0.001*
Sex			
M	1193 (48)	1652 (57)	*P* < 0.001
F	1272 (52)	1261 (43)	
Grade			
1	255 (10)	349 (12)	*P* < 0.001
2	1614 (67)	2234 (78)	
3/4	550 (23)	285 (10)	
Lymphovascular invasion			
‐	1785 (72)	2215 (76)	*P* = 0.002
+	680 (28)	698 (24)	
Perineural invasion			
‐	2292 (93)	2666 (92)	*P* = 0.047
+	173 (7)	247 (9)	
Stage			
I	219 (9)	307 (11)	*P* = 0.008
II	948 (39)	1006 (35)	
III	1286 (52)	1556 (54)	
Lymph nodes removed			
>12	1458 (60)	1262 (45)	*P* < 0.001
<12	958 (40)	1542 (55)	
ECOG			
0‐1	2203 (89)	2607 (90)	*P* = 0.883
2+	262 (11)	306 (11)	
Adjuvant chemotherapy			
No	1332 (54)	1374 (47)	*P* < 0.001
Yes	1133 (46)	1539 (53)	

Abbreviations: present: “+”; absent: “‐”; female: F; male: M; IQR: interquartile range.

* Wilcoxon‐rank sum.

Compared with left, right‐sided tumors were significantly more frequent among women (52% versus 43%), more likely to be high grade (23% versus 10%), stage II (39% versus 35%), and have lymphovascular invasion. Patients with right‐sided cancers were older (70 versus 66 years), more likely to have >12 nodes removed (60% versus 45%), and less likely to receive adjuvant chemotherapy (none, 54% versus 47%).

### 3.2 Relapse‐free survival

Kaplan Meier survival analyses demonstrated a positive prognostic effect of right‐ sidedness in stage II tumors; these patients experienced significantly better 5‐year RFS compared with those with left‐sided tumors (Table 2). Among stage III patients, 5‐year RFS was similar between right and left origin. A multivariate Cox proportional hazards model was established for stage II tumors (Table 3), and the proportional hazard assumption holds for all covariates. Among 1954 patients with stage II tumors, those with right‐sided tumors experienced better RFS compared with left [HR 0.73 (95% CI 0.59‐0.91), *P* = 0.006]. Lymphatic or vascular invasion, and less than 12 nodes harvested, were each independently associated with worsened survival. Neither the receipt of adjuvant chemotherapy nor tumor grade influenced RFS among patients with stage II tumors. For stage III tumors, a stratified multivariate Cox model was used to allow for tumor grade and adjuvant chemotherapy with nonproportionality (Table 3). Among stage III patients, right‐versus left‐sidedness was not prognostic, while lymphatic or vascular or perineural invasion, worse performance score, and the removal of less than 12 lymph nodes, all conferred a decreased RFS.

**Table 2:** Kaplan Meier survival analyses of 5‐year relapse‐free survival and 5‐year overall survival after relapse in patients with stage I‐III colon cancer

	5‐Year Relapse Free Survival (%)				
	N	R	L	R vs L HR (95% CI)	*P*‐Value
Stage I	526	94.46 (90.20‐96.90)	93.51 (90.02‐95.81)	0.79 (0.40‐1.55)	0.487
Stage II	1946	85.06 (82.56‐87.23)	78.03 (75.27‐80.53)	0.65 (0.53‐0.80)	<.0001
Stage III	2840	63.94 (61.18‐66.55)	66.52 (64.06‐68.85)	1.08 (0.96‐1.22)	0.214
	5Y Overall SAR (%)			R vs L HR (95% CI)	*P*‐Value
Relapsed II	384	10.62 (6.26‐16.30)	21.39 (16.20‐27.06)	1.53 (1.22‐1.90)	0.0002
Relapsed III	1012	5.34 (3.29‐8.10)	12.34 (9.57‐15.49)	1.42 (1.25‐1.62)	<.0001

**Table 3:** Cox proportional hazards model of relapse‐free survival among stage II (*N* = 1954) and stage III (*N* = 2842) colon cancer

	Stage II (*N* = 1954)			Stage III (*N* = 2842)		
RFS Variable	HR	95% CI	*P*‐Value	HR	95%CI	*P*‐Value
R vs L	0.73	(0.588‐0.914)	0.006	1.03	(0.905‐1.181)	0.624
Grade 3/4 vs 1/2	0.96	(0.688‐1.340)	0.812	~ *	~	~ *
M vs F	1.11	(0.898‐1.366)	0.340	1.04	(0.911‐1.175)	0.596
Age	1.00	(0.988‐1.007)	0.572	1.00	(0.992‐1.004)	0.493
LVI + vs ‐	1.44	(1.087‐1.903)	0.011	1.63	(1.420‐1.861)	<.0001
PNI + vs ‐	1.33	(0.888‐1.979)	0.168	1.39	(1.156‐1.673)	0.001
LN removed						
<12 vs ≥12	1.90	(1.527‐2.367)	<.0001	1.24	(1.083‐1.408)	0.002
ECOG status 0/1 vs 2+	1.01	(0.814‐1.249)	0.939	0.87	(0.758‐0.998)	0.047
AC No vs. Yes	1.04	(0.802‐1.340)	0.785	~ *	~	~ *

* HR and *P*‐value were not generated for stratification variables as the proportionality assumption was not satisfied. Abbreviations: variables stratified in the Cox model: “~”; present: “+”; absent: “‐”; adjuvant chemotherapy: AC; confidence interval: CI; female: F; hazard ratio: HR; left: L; lymph node: LN; lymphovascular invasion: LVI; male: M; perineural invasion: PNI; relapse free survival: RFS; right: R.

### 3.3 Overall survival after relapse (SAR)

Overall survival analyses were conducted among the patients in the original cohort who developed a local, regional, or distant relapse (Tables 2 and 4). Results showed that the favorable prognostic effect of right‐sided stage II patients was reversed, and 5‐year overall SAR was significantly inferior for right versus left‐sided tumors [HR 1.53 (95% CI 1.22‐1.90), *P* <0.001]. Among relapsed stage III tumors, 5‐year SAR was similarly inferior [HR 1.42 (95% CI 1.25‐1.62), *P* < 0.001].

**Table 4:** Cox proportional hazards model of overall survival after relapse among stage II (*N* = 384) and stage III (*N* = 1012) colon cancer patients who relapsed

	Stage II (*N* = 384)			Stage III *(N* = 1012)		
Overall SAR Variables	HR	95%CI	*P‐*Value	HR	95% CI	*P*‐Value
R vs L	1.34	(1.073‐1.681)	0.010	1.31	(1.14‐1.50)	0.0001
Grade 3/4 vs 1/2	1.17	(0.814‐1.675)	0.399	~ *	~	~ *
M vs F	1.11	(0.898‐1.372)	0.334	0.99	(0.867‐1.134)	0.903
Age	1.02	(1.011‐1.030)	<.0001	1.02	(1.014‐1.028)	<.0001
LVI + vs ‐	1.11	(0.841‐1.471)	0.457	1.08	(0.938‐1.246)	0.281
PNI + vs ‐	1.12	(0.742‐1.691)	0.589	1.11	(0.919‐1.349)	0.273
LN Removed <12 vs ≥12	1.08	(0.869‐1.348)	0.479	1.01	(0.886‐1.162)	0.836
ECOG status** 0/1 vs 2+	1.07	(0.862‐1.336)	0.528	0.78	(0.673‐0.901)	0.0008
AC No vs. Yes	1.08	(0.840‐1.388)	0.548	1.27	(1.065‐1.514)	0.008

* HR and *P*‐value were not generated for stratification variables as the proportionality assumption was not satisfied. Abbreviations: variables stratified in the Cox model: “~”; present: “+”; absent: “‐”; adjuvant chemotherapy: AC; confidence interval: CI; female: F; hazard ratio: HR; left: L; lymph node: LN; lymphovascular invasion: LVI; male: M; perineural invasion: PNI; relapse free survival: RFS; right: R; survival after relapse: SAR.

** At diagnosis of early stage disease.

### 3.4 Survival and therapy with biologics

**Figure 1:**
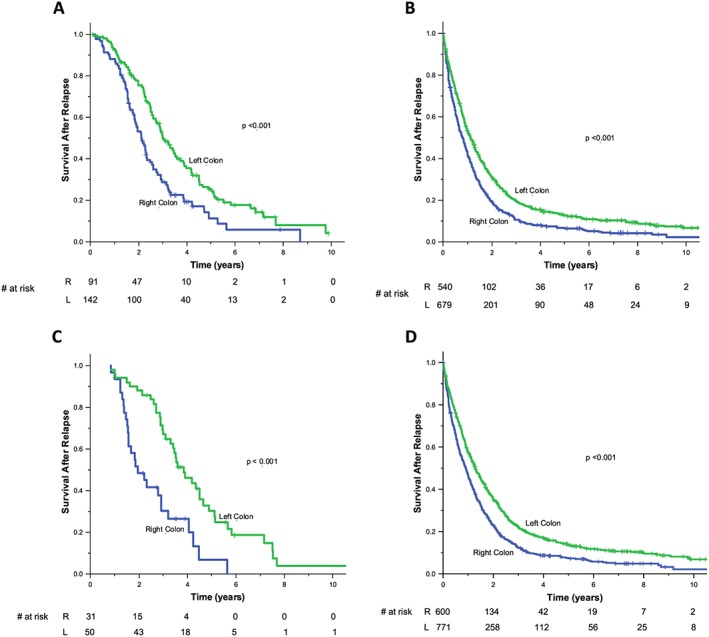
Kaplan Meier overall survival analysis by tumor sidedness and receipt of therapy. Left‐sided tumours demonstrated superior survival when treated with bevacizumab [HR 0.58 (95% CI 0.43‐0.78)] (Panel A) and when not treated by bevacizumab [HR 0.73 (95% CI 0.64‐0.82)] (Panel B). There was no association between bevacizumab and sidedness and survival (*P* = 0.195). Left‐sided *KRAS* wt tumors had superior survival when treated with EGFRIs [HR 0.38 (95% CI 0.22‐0.64)] (Panel C) and in the absence of treatment by EGFRIs [HR 0.72 (95% CI 0.64‐0.80)] (Panel D). *P*‐values in the figures indicate the comparison of sidedness in the treatment groups. There was a significant interaction between sidedness and EGFRI therapy on overall survival (*P* = 0.017), with a greater effect seen left‐sided tumors

Kaplan Meier median OS estimates of patients with relapsed disease are shown, based on tumor sidedness and receipt of bevacizumab (Figure 1, panel A and B) and EGFRI therapy in *KRAS* wild type (wt) tumors (Figure 1, panel C and D). Significant differences in survival outcomes were observed by tumor sidedness in all treatment groups. Patients with left‐sided tumors demonstrated superior survival irrespective of whether they received bevacizumab [HR 0.58 (95% CI 0.43‐0.78) *P* = 0.0003] or not [HR 0.73 (95% CI 0.64‐0.82) *P* < 0.0001]. A test of interaction did not reveal any association between bevacizumab therapy and sidedness (*P* = 0.195). Left‐sided, *KRAS* wt patients treated with EGFRI's [HR 0.37 (95% CI 0.22‐0.63)] and without EGFRIs [HR 0.72 (95% CI 0.64‐0.80)] also had superior outcomes (Wald test, *P* < 0.0001); however, a test of interaction revealed a significant interaction between sidedness and EGFRI therapy and OS (*P* = 0.017).

## 4 DISCUSSION

In this study, patients with right‐sided stage II colon cancer had improved RFS in a multivariate model, and no prognostic effect was observed in stage III tumors. When survival was considered after relapse, right‐sided tumors had inferior OS compared with left‐sided tumors in initially stage II and stage III tumors. Right‐sided outcomes were inferior irrespective of type of palliative systemic therapy, and a test of interaction revealed an association between sidedness and survival with EGFRi therapy in *KRAS* wt tumors.

The favorable prognostic effect of sidedness in stage II CCa demonstrated in this study is similar to that demonstrated in prior studies. Right versus left‐sided origin was associated with a HR of 0.73 (0.59‐0.91), and other studies have demonstrated a similar effect (HRs, 0.92, 0.89, and 0.91 respectively).^5^, ^7^, ^8^ High tumor grade was more frequent in stage II right‐sided tumors but was not independently prognostic; poorly differentiated, stage II tumors are more frequently MSI high. Prior studies demonstrated that right‐sided stage II tumors have an improved prognosis, an effect almost entirely due to the MSI population,^9^ which is significantly more frequent in stage II than stage III malignancies.^19^

A significant finding of the current study was the lack of prognostic effect of sidedness among stage III patients, which calls into question whether sidedness is a pertinent consideration in the adjuvant treatment of node‐positive tumors. Results of a prior prospective trial of stage III CCa also demonstrated the lack of prognostic effect of sidedness on RFS,^9^ and more recent data, NSABP C‐07^16^ and PETTAC8,^17^ both reported that lack of prognostic effect of sidedness in stage III tumors; sidedness was only prognostic if tumors relapsed. While retrospective studies have demonstrated inferior OS of patients with right‐sided tumors in the stage III setting [HR, 1.12 (95% CI, 1.06‐1.18),^5^ HR 1.06 (95% CI 1.02‐1.11),^8^ HR 1.12 (95% CI 1.09‐1.15)],^7^ propensity score matching to adjust for patient characteristics among 91,416 SEER patients found no prognostic effect of sidedness in stage III CCa for OS or cancer specific survival.^15^ In the current study, RFS was used to evaluate the prognostic effect of sidedness in the early stage while OS was considered among patients with relapsed tumors, which provides a deeper understanding of survival compared with the population‐based studies. While right‐sided stage III tumors may not have a higher risk of relapse, they do experience inferior outcomes if they recur, and sidedness becomes an important treatment consideration.

In the advanced setting after relapse, sidedness was a strong prognostic factor, with right‐ sidedness associated with an HR of 1.34 (95% CI 1.07‐1.68) in stage II and 1.31 (95% CI 1.14‐1.50) in stage III patients. The size of the prognostic effect was similar to that seen in stage IV disease in SEER cohorts, where HRs for right‐sided presentation were reported as 1.22 (95% CI 1.16‐1.28)^8^ and 1.25 (95% CI 1.22‐1.27)^7^ in 2 US studies, 1.12 (95% CI 1.02‐1.23) in a German study,^2^ and 1.57 (95% CI 1.44‐1.72) in an Australian population‐based analysis.^11^ In an analysis of 3 prospective trials of patients with advanced colorectal cancer, HRs for right‐sidedness all showed a significant detrimental effect on progression‐free and OS.^10^ Recent analysis of multiple randomized controlled trials of EGFRI therapy^20^, ^21^, ^22^ has all confirmed the adverse prognostic effect of right‐sidedness, raising the hypothesis that the differing embryologic origin of the right versus left gut confers inherent differences in tumor biology. Others have previously explored the molecular characteristics of proximal tumors, which were strongly associated with high *BRAF* signature scores and contributed to inferior outcomes even among tumors that were microsatellite‐stable, *BRAF*, and *KRAS* WT, and may partially explain the poor survival of patients with right‐sided tumors independent of known prognostic factors.^9^

The current study demonstrated the prognostic effect of sidedness in the advanced stage irrespective of bevacizumab or EGFRI therapy. However, a test of interaction demonstrated a significant association between sidedness and EGFRI therapy, with *KRAS* WT left‐sided EGFRI‐treated tumors experiencing a significantly improved survival versus those with right‐sided tumors. Similar observations were made in randomized clinical trials. Post‐hoc analyses of multiple trials of first, second, and third line prospective trials of EGFRI therapy,^20^, ^21^, ^22^, ^23^, ^24^ demonstrated the predictive value of tumor location on OS, and potential interaction with the biologic agents bevacizumab and EGFRI. The current study is among the first population‐based studies to demonstrate an association between sidedness and responsiveness to EGFRI therapy.

### 4.1 Limitations

The major limitation of this study, which is one shared with numerous population‐based studies, is the lack of detailed molecular marker status. While sidedness is prognostic, it is very likely a surrogate for differences in the distribution of molecular factors or cancer subtypes between the right and left side of the colon. Such biomarkers would likely offer a more precise means to define prognosis and predict response to planned therapy than sidedness does. As All‐RAS and BRAF testing only became standard in advanced disease after 2013, this data is not available in this study which included only patients until 2009. Similarly, MSI status was not prospectively collected for the patients in this cohort. Nevertheless, multiple studies have demonstrated the prognostic effect of sidedness in the advanced setting that is independent of tumor genotype and Consensus Molecular Subtypes (CMS).^25^

## 5 CONCLUSIONS

The study shows that right‐sidedness confers a favorable prognosis to stage II CCa but is not prognostic of relapse in stage III colon cancer. Right‐sidedness is a strong adverse prognostic factor among patients who relapse and may influence the selection of therapy. While more accurate biomarkers are awaited, sidedness should be considered as a relevant stratification factor in studies of both early and advanced disease, as imbalances between study arms can significantly affect study outcomes. Previously validated prognostic variables remain relevant and should continue to be incorporated in multivariate analysis and reflect the complexity of CCa disease biology.

## AUTHOR CONTRIBUTIONS

All of the authors contributed to the conception, data analysis, and writing of this paper.

Conceptualization: Kennecke, Yin, Davies, Cheung, Lee‐Ying

Data Curation: Kennecke, Yin, Speers, Cheung, Lee‐Ying

Formal Analysis: Kennecke, Yin, Speers, Cheung, Lee‐Ying

Methodology: Kennecke, Yin, Cheung, Lee‐Ying

Writing – review and editing: Kennecke, Yin, Davies, Speers, Cheung, Lee‐Ying

Writing –original draft: Kennecke, Yin, Davies, Cheung, Lee‐Ying

## FUNDING

This work was supported by the BC Cancer Foundation (BCCF). The BCCF did not provide any oversight nor did it have any influence in how the research was conducted.

## CONFLICTS OF INTEREST

None of the authors have any conflicts of interest related to this paper. H.F.K. has served in an advisory role to Amgen Canada and Ipsen Canada. J.M.D. has served in an advisory role to Amgen, Bayer, Ipsen, and Spier. R.L.H. has served in an advisory role to Bayer and BMS. Y.Y., C.H.S., and W.Y.C. have no conflicts of interest to report.
